# Childhood Maltreatment and Creativity among Chinese College Students: A Serial Mediation Model

**DOI:** 10.3390/jintelligence11040058

**Published:** 2023-03-24

**Authors:** Wenfu Li, Shuai Zhang, Hao Lin, Keke Zhang, Xiaolong Zhang, Jingting Chen, Fangfang Xu, Chuanxin Liu

**Affiliations:** 1 School of Mental Health, Jining Medical University, Jining 272067, China; 2 College of Chinese and Western Medicine, Jining Medical University, Jining 272067, China; 3 Labor Union, Jining Medical University, Jining 272067, China

**Keywords:** childhood maltreatment, creativity, cognitive flexibility, self-efficacy, college students

## Abstract

Creativity plays a very crucial impact on our cultural life and has also been important to the improvement of human civilization. Numerous studies have indicated that family circumstance plays an important role in the development of individual creativity. However, little is known about the mediating mechanisms underlying the association between childhood maltreatment and creativity. This study intended to explore the serial multiple mediation model in which undergraduates’ cognitive flexibility and self-efficacy were proposed to mediate the potential influence of childhood maltreatment on their creativity. Participants were 1069 undergraduates (573 males and 496 females, mean age was 20.57 ± 1.24 years ranging from 17 to 24) from a university in Shandong Province, China. Participants were required to complete an internet survey including the Short Form of Childhood Trauma Questionnaire (CTQ-SF), General Self-Efficacy Scale (GSES), Cognitive Flexibility Inventory (CFI), and Williams Creativity Aptitude Test (WCAT). Serial multiple mediation analysis and the bootstrap method were used to investigate the mediation effects of cognitive flexibility and self-efficacy. The results showed that childhood maltreatment indirectly influenced undergraduates’ creativity through three indirect paths: childhood maltreatment→cognitive flexibility→creativity, childhood maltreatment→self-efficacy→creativity, and childhood maltreatment→cognitive flexibility→self-efficacy→creativity. The ratios of the total indirect effects and branch-indirect effects to the total effects were 92.73%, 34.61%, 35.68%, and 22.44%, respectively. These results indicated that cognitive flexibility and self-efficacy could completely mediate the potential impact of childhood maltreatment on individuals creativity.

## 1. Introduction

Creativity plays a very critical impact on our cultural life and has also been crucial to the improvement of human civilization (Takeuchi et al. 2010). Furnham and Bachtiar (2008) proposed the idea of creativity, which was the ability that was designed to generate a product or idea that was characteristic of novel and useful. Given the crucial role of creativity, a great number of studies have been conducted to investigate the possible mechanisms that are conductive to creativity. Numerous studies have found that environment factors are one of the most important variables to influence creativity (Ma 2009; Onarheim and Friis-Olivarius 2013). As one of the early exposed environments, family circumstances played an important role in the development of individual creativity. Various cognitive functions such as creativity were influenced by the family environment and parenting style (Chan 2005; Si et al. 2020). If there is an association between family cultivating and creativity, one can speculate that childhood maltreatment has an important effect on an individual’s capacity to achieve excellent creative accomplishment. However, the relationship between childhood maltreatment and individual creativity has not been clarified so far. This study aimed to explore the relationship between childhood maltreatment and creativity and further investigate the mediation mechanism of cognitive flexibility and self-efficacy.

### 1.1. Childhood Maltreatment and Creativity

Childhood maltreatment, defined as adverse events including physical abuse, emotional abuse, sexual abuse, physical neglect, and emotional neglect against children occurring prior to the age of 18 years, was one of the risk factors for potential or actual hurt to mental and physical health (Bernstein et al. 2003). Childhood abuse and neglect were serious problems that related to public health, legal issues, and society harmony, and caused considerable adverse influences on the people’s physiological and psychological growth (Capusan et al. 2021; Liu et al. 2022). They were also common and severe issues among Chinese university students (Fu et al. 2018). Numerous studies have documented a significant relationship between childhood maltreatment and mental problems, as well as behaviors problems (Kadivari et al. 2022; Lamela and Figueiredo 2018; McRae et al. 2022).

Childhood maltreatment not only predicted crime, domestic violence, and antisocial personality (Ducci et al. 2008; Fox et al. 2015; Pournaghash-Tehrani and Feizabadi 2009), but also influenced executive function, cognitive flexibility, working memory, and openness to experience (Bernardes et al. 2020; Chiasson et al. 2021; Fletcher and Schurer 2017; Spann et al. 2012), which were related with creativity (Benedek et al. 2014; Sun et al. 2018; Wu and Koutstaal 2020; Xing et al. 2019). Recent research indicated that childhood adverse experience, such as low family socioeconomic status and poor parent–child relationships, was negatively related with social creativity (Zhang et al. 2018). Other studies also found that creative individuals often had a warmer childhood environment and harmonious family (Chan 2005; Zaeske et al. 2022). A recent study indicated that parental warmth and parental rejection were positively related with creativity (Guo et al. 2021). Additionally, two recent studies have found a positive relationship between childhood maltreatment and malevolent creativity (Jia et al. 2020; Li et al. 2022), which is defined as the ability to generate creative ideas to harm others intentionally (Cropley et al. 2014; Gill et al. 2013; Hao et al. 2016). For example, Jia et al. (2020) demonstrated that childhood neglect and Dark Triad personality traits were positively associated with malevolent creativity. Another study further found that childhood maltreatment and aggression were positively related with malevolent creativity (Li et al. 2022). However, it was also indicated that maltreated individuals developed intact, or stress-adapted skills for solving problems in adverse situations (Ellis et al. 2022). Therefore, based on these close relationships between adverse experience in early life and social creativity, as well as malevolent creativity, it is meaningful to investigate whether and how childhood maltreatment influences individual creativity.

### 1.2. Cognitive Flexibility as a Mediator

Cognitive flexibility, one of many crucial factors influencing individual creativity (Guilford 1967), can be defined as the capacity to switch thinking sets to adapt to the changing environmental stimulus (Dennis and Vander Wal 2010). Cognitive flexibility theory proposed by Spiro and Jehng (1990) defined cognitive flexibility as “the ability to spontaneously restructure one’s knowledge, in many ways, in adaptive response to radically changing situational demands…” (p. 165). From a general view, the findings of studies focusing on cognitive control, attention, associative process, executive function, prefrontal cortex function, and Rapid Eye Movement (REM) sleep (Beaty et al. 2014; Dietrich 2004; Dorfman et al. 2008; Stickgold and Walker 2004; Zabelina and Robinson 2010) collectively suggested that creativity was associated with cognitive flexibility. Additionally, high creative participants, compared with less creative participants, performed well in the levels of flexible cognitive control (Zabelina and Robinson 2010). Recent researches also found that cognitive flexibility was positively correlated with creative problem-solving (Wu and Koutstaal 2020; Zuo et al. 2019). Therefore, we hypothesize that cognitive flexibility positively predict individuals creativity.

A burgeoning study about exposure during early life to childhood maltreatment showed that, in addition to mental or behaviors problems (Kadivari et al. 2022; Lamela and Figueiredo 2018; McRae et al. 2022), childhood maltreatment was related to a range of cognitive function impairments and psychological disorders (Su et al. 2019; Xiao et al. 2021). Associations between maltreatment experience and executive function have been certified consistently in adolescents and adults (Letkiewicz et al. 2021; Mothes et al. 2015). For example, Spann et al. (2012) found that both physical abuse and neglect were related to a reduction in cognitive flexibility in 12–17-years old adolescents. Similarly, Kalia and Knauft (2020) further found childhood adversity was correlated with decreased cognitive flexibility in adulthood. Thus, it is reasonable to hypothesize that childhood maltreatment negatively predicts cognitive flexibility.

Given the close relationship between childhood maltreatment and cognitive flexibility (Kalia and Knauft 2020; Spann et al. 2012), and cognitive flexibility and creativity (Wu and Koutstaal 2020; Zuo et al. 2019), cognitive flexibility might be considered to be an important variable in the hypothesized connection between childhood maltreatment and individual creativity. The Dual Pathway to Creativity Model (DPCM) proposed that dispositional or situational factors might affect creativity through cognitive flexibility or cognitive persistence pathway, and any characteristics or state that reduced either cognitive flexibility or cognitive persistence would hinder individuals creativity (Baas et al. 2013). Indeed, empirical research indicated that cognitive flexibility mediated the link between early life maltreatment and psychological characteristic (Kalia et al. 2020). Therefore, it is reasonable that the relationship between childhood maltreatment and creativity might be mediated by the effect of cognitive flexibility.

### 1.3. Self-Efficacy and Creativity

Self-efficacy was the ability to judge about whether or not one has the capability to complete certain types of performance that were related to the individual (Puente-Díaz 2016). The social cognitive theory putted forward that self-efficacy played an important motivational role in creative thinking (Bandura 1997). Previous empirical studies indicated that self-efficacy influenced individual creativity positively (Daher et al. 2021). For instance, Tamannaeifar and Motaghedifard (2014) found that self-efficacy had a significant positive influence on the creativity of university students. Other research also emphasized that self-efficacy was a crucial predictor of creativity (Waheed and Dastgeer 2020). In a recent meta-analysis research, Haase et al. (2018) synthesized 60 effect sizes from 41 articles, which explored the association between self-efficacy and creativity, and revealed that self-efficacy was positively correlated with multifaceted creativity measures, such as self-rated creativity and divergent thinking. Thus, the present research proposes that self-efficacy positively correlates with self-rated creativity.

Other research also found a similar relationship between childhood maltreatment and self-efficacy. For instance, Lu et al. (2017) investigated the association among childhood maltreatment, self-efficacy, and abstinence motivation, and found that childhood maltreatment was negatively correlated with self-efficacy. Additionally, Soffer et al. (2008) found that childhood emotional neglect was associated with the lower level of self-efficacy in undergraduates. These findings indicated that childhood maltreatment was an important negative predictor of both cognitive flexibility and self-efficacy. 

Meanwhile, social cognitive theory (Bandura 1997) suggested that the successful experience was a major source of self-efficacy. However, individuals who experienced childhood abuse or neglect had more denial from caregiver and failure experience, and this may have led to low levels of self-efficacy (Adjorlolo et al. 2017). Therefore, based on the association between childhood maltreatment and self-efficacy (Lu et al. 2017; Soffer et al. 2008), and self-efficacy and creativity (Daher et al. 2021; Waheed and Dastgeer 2020), we hypothesize that self-efficacy might mediate the influence of childhood maltreatment on creativity.

### 1.4. A Serial Multiple Mediator Model

Furthermore, people who have a high level of cognitive flexibility confront difficulties and are more flexible with what they could do with these troubles and more confident in themselves; people who have a low level of cognitive flexibility become overwhelmed by not knowing what to do and are less confident about their capability. It is essential for individuals to be flexible and to handle changes and difficulties quickly. An empirical study revealed that cognitive flexibility was positively related to self-efficacy (Esen et al. 2017). Bandura, who proposed the self-efficacy was the portion of cognitive flexibility, pointed out that the more cognitive flexibility people have, the higher their level of self-efficacy (Esen et al. 2017). Other studies also revealed that there was a meaningful association between cognitive flexibility and self-efficacy (Demirtaş 2020). Accordingly, the previous findings indicated that the more cognitive flexibility, the more self-efficacy (Esen et al. 2017). Therefore, we hypothesize that childhood maltreatment might influence self-efficacy through the mediated role of cognitive flexibility. 

Additionally, given the potential mediation effect of cognitive flexibility and self-efficacy between childhood maltreatment and creativity, we assume that childhood maltreatment might influence creativity through the serial multiple mediation effects of cognitive flexibility and self-efficacy. 

### 1.5. The Current Study

The above discussions indicated obviously that childhood maltreatment, cognitive flexibility, self-efficacy, and individuals creativity were correlated with each other. In other words, the current study intended to investigate the serial multiple mediation roles of cognitive flexibility and self-efficacy between the influence of childhood maltreatment on creativity. Based on the research reviewed above, the present study was conducted to investigate the potential patterns in (1) the influence of childhood maltreatment on creativity; (2) the mediating role of cognitive flexibility and self-efficacy in the association between childhood maltreatment and creativity respectively; (3) the serial multiple mediation effects of cognitive flexibility and self-efficacy in the relationship between childhood maltreatment and individual creativity.

## 2. Materials and Methods

### 2.1. Participants

This study enrolled 1120 undergraduates from universities in Shandong Province, China. All participants volunteered for the online questionnaire survey on the website www.wjx.cn. All the questionnaires used in the present research were revised or established using a standard procedure. All the items were in a clear and easily understandable Chinese version. It takes about five minutes to complete the survey. The data of 19 participants who completed the questionnaires with simply repeated answers or fixed patterns (e.g., participants completed a four-point Likert scale with 1, 2, 3, and 4 in turn), and 32 participants whose reaction time was beyond three standard deviations, were omitted from further analysis. Finally, the present study included 1069 participants (573 males and 496 females, mean age was 20.57 ± 1.24 years ranging from 17 to 24), including birthplace in cities (n = 699, 65.4%) and rural areas (n = 370, 34.6%), and only child (n = 452, 42.3%) and not an only child (n = 617, 57.7%). The research program was approved by the local Ethics Committee.

### 2.2. Measures

#### 2.2.1. Short Form of Childhood Trauma Questionnaire (CTQ-SF)

The Chinese version (Zhao et al. 2005) of CTQ-SF (Bernstein et al. 2003) was used, which consisted of 28 items (included 25 clinical items and 3 validity items) and assessed childhood abuse and neglect histories that happened before 18 years of age. Each item asked about maltreatment experience in childhood and adolescence and was graded on a five-point Likert scale with five answer options ranging from Never to Always. The CTQ-RS has five clinical factors: physical, emotional, and sexual abuse, and physical and emotional neglect. Each type of factor was represented by five items. The sample items and the definitions of abuse and neglect can be found in previous researches (Bernstein et al. 1994; Bernstein et al. 2003). The score of CTQ-SF was equal to the sum of 25 clinical items. The higher the score of CTQ-SF, the more serious the childhood maltreatment was. This Chinese version has satisfactory reliability and validity which have been empirically derived (Zhao et al. 2005). The Cronbach’s alpha coefficient in the present study was 0.920.

#### 2.2.2. General Self-Efficacy Scale (GSES)

The Chinese version of the GSES was used in the present research, which was adapted by Zhang and Schwarzer (1995). The GSES consisted of 10 items and measured the participants’ beliefs or expectation about the capacity to finish tasks on their own. Each item was rated on a four-point Likert-type scale with four reaction options ranging from Never True to Very Often True. The score of GSES was equivalent to the sum of all 10 items. The higher the score of GSES, the stronger the ability of self-efficacy was. This scale has high reliability and validity (Zhang and Schwarzer 1995). The Cronbach’s alpha coefficient was 0.835.

#### 2.2.3. Cognitive Flexibility Inventory (CFI)

The Chinese version (Wang et al. 2016) of the CFI (Dennis and Vander Wal 2010) was used to evaluate the ability of cognitive flexibility to successfully overcome or displace inappropriate thoughts with more appropriate and concordant thinking. This scale consisted of 20 self-report items that were rated on a five-point Likert-type scale with five forced-choice options ranging from Never to Very Often. The score of CFI was calculated with the sum of numerical response values. Higher scores implied the greater cognitive flexibility, while lower scores represented the greater cognitive rigidity (Dennis and Vander Wal 2010). The reliability and validity with the Chinese participants of this questionnaire were satisfactory. The Cronbach’s alpha coefficient of CFI was 0.885.

#### 2.2.4. Williams Creativity Aptitude Test (WCAT)

The Chinese version developed by Lin and Wang (1994) was utilized to measure creativity. This test included 50 self-assessment items. Participants were required to rate on a three-point Likert scale ranging from totally disagree to totally agree. The score of WCAT was the sum of numerical response values of 50 items (eight items were required reverse coding firstly). The higher score of WCAT represented the higher creative potential. The split half reliability and test–retest reliability of this Chinese version were satisfactory (Hwang et al. 2007). The Cronbach’s alpha coefficient of WCAT in the present research was 0.893.

### 2.3. Statistical Analysis

The SPSS and PROCESS V3.3 developed by Hayes (2013), a free-to-use macros for SPSS used to calculate the mediation and moderation effects, were utilized to analyze the data. Pearson correlation test was used to calculate the relationship between variables. Template model six of PROCESS V3.3, which tested the serial multiple mediators model, was used to compute the serial mediating effects of cognitive flexibility and self-efficacy. PROCESS V3.3 calculated the total effects, direct effects, and indirect effects with a 95% bootstrap confidence interval based on 5000 resamples. The *p* = 0.05 was used as statistically significant.

## 3. Results

### 3.1. Common Method Bias Test

We used Harman’s single factor test to examine the common method bias and imput all the items of CTQ-SF, GSES, CFI, and WCAT into the un-rotated exploratory factor analysis. The results revealed that 18 components with initial eigenvalues greater than 1 were extracted from the factorial analysis. The explanation rate of the component with maximum initial eigenvalue was 18.47%, which did not exceed the critical value of 40%. These results indicated that there was no obvious common method bias in the present research.

### 3.2. Descriptive Statistical Analysis

Descriptive statistical analysis results are shown in Table 1. Characterization of the Skewness and Kurtosis indicated that the score of childhood maltreatment, cognitive flexibility, self-efficacy, and creativity basically fitted the normal distribution (Hancock et al. 2010). Given the large sample size, the raw data were used in the following statistical analysis followed (Tabachnick and Fidell 2007). 

### 3.3. Correlation Analysis

The results of the Pearson correlation analysis of variables are shown in Table 2. The results indicated that the score of CTQ-SF was negatively related to GSES, CFI, and WCAT respectively, while the scores of GSES, CFI, and WCAT were positively correlated with each other. 

### 3.4. Test of the Serial Multiple Mediation Effects of Cognitive Flexibility and Self-Efficacy

The serial multiple mediation effects of cognitive flexibility and self-efficacy were analyzed using PROCESS V3.3 followed Hayes (2013). In the multiple regression analysis, the score of WCAT was the dependent variable and the score of CTQ-SF was the independent variable. The scores of CFI and GSES were the mediating variables. Gender, age, urban or rural areas, and singletons or non-singletons were the control variables to reduce the possible effects of the demographic variables on the mediation analysis. The unstandardized regression coefficient was calculated to reduce the type I errors caused by the data distribution. The significant level of path coefficients and 95% confidence interval were computed using multiple regression analysis and bootstrap method. The mediating effects were statistically significant, if the 95% bias-corrected bootstrap confidence interval did not contain zero (Hayes 2013). The standardized regression coefficients were calculated in the above model using the standardized data.

**Figure 1:**
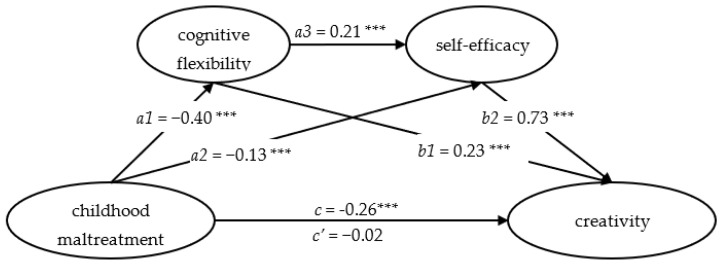
The serial multiple mediation of attachment anxiety and interpersonal relationship between adverse childhood experience and mobile phone use. Note: path coefficients were shown in unstandardized regression coefficient. *** *p* < 0.001.

The results of serial multiple mediation analysis are shown in Table 3 and Figure 1. The results revealed that the total effect of childhood maltreatment on creativity, and the total indirect effects of cognitive flexibility and self-efficacy, was statistically significant, while the direct effect was not significant. Therefore, cognitive flexibility and self-efficacy played a complete mediating role between childhood maltreatment and creativity.

Furthermore, all three indirect effects paths were statistically significant based on the 95% bootstrap confidence interval. That is to say, the childhood maltreatment influenced creativity through three indirect paths: childhood maltreatment→cognitive flexibility→creativity, childhood maltreatment→self-efficacy→creativity, and childhood maltreatment→cognitive flexibility→self-efficacy→creativity. The total effect was −0.26 (*t* = −13.42, *p* < 0.001), while the direct effect was −0.02(*t* = −0.79, *p* = 0.43) and the total indirect effect was −0.24. The three branch-indirect effects were −0.09 (a1 × b1), −0.09 (a2 × b2), and −0.06 (a1 × a3 × b2), respectively. The ratios of total indirect effect and branch-indirect effects to total effect were 92.73%, 34.61%, 35.68%, and 22.44%, respectively. 

## 4. Discussion

The present study intended to explore the potential mediating mechanisms underlying the relationship between childhood maltreatment and creativity. The principal finding was that childhood maltreatment, at least as reported retrospectively, was associated with self-reported creativity indirectly through the mediation effects of cognitive flexibility and self-efficacy, and their serial multiple mediating role. This result provided new psychological perspectives to minimize the negative influence of childhood maltreatment, strengthen cognitive flexibility and self-efficacy, and cultivate the creative thinking of college students. 

### 4.1. The Relation between Childhood Maltreatment and Creativity

Consistent with that hypothesis, childhood maltreatment was found to be negatively correlated with self-report creativity among Chinese college students. In accordance with our result, other studies also indicated that early adverse experience was associated with different measures of creativity. For example, Zhang et al. (2018) investigated the influence of family socioeconomic conditions and parent–child relations on social creativity, and found that both socioeconomic conditions and parent–child relations were positively associated with social creativity. Gralewski and Jankowska (2020) further found that both parental child acceptance and autonomy were positively correlated with high school students’ creative ability and self-belief. The children who had experienced maltreatment usually have impaired executive function, lower cognitive flexibility, poor working memory, and low level of openness to experience (Bernardes et al. 2020; Chiasson et al. 2021; Fletcher and Schurer 2017; Spann et al. 2012). These cognitive functions and personality traits facilitated the development of individual creativity (Beaty et al. 2014; Miller and Tal 2007; Zabelina and Robinson 2010). The present result further indicated that childhood maltreatment has a strong negative influence on latter creativity.

Furthermore, the childhood maltreatment experience not only hindered the development of benevolent creativity, but also promoted the dark side of creativity, commonly called malevolent creativity (Cropley et al. 2014). The influence of malevolent creativity was mainly shown in the domain of terrorism and law-breaking conduct (Cropley and Cropley 2011; Gill et al. 2013). Interestingly, a recent study found that childhood neglect positively predicted the malevolent creativity of undergraduate students (Jia et al. 2020). Another study also found that childhood maltreatment and aggression were positively related with malevolent creativity (Li et al. 2022). Thus, individuals who have experienced more neglect or abuse in child’s period were likely to pursue more malevolent creativity and less benevolent creativity. Parents should be more attentive to their children’s needs and healthy growth to prevent malevolent creativity from happening. Notably, burgeoning studies on the exposure during childhood to harsh, unpredictable environments indicated that, in addition to a consequential lower level on a range of cognitive performance, such as intelligence, language, and executive functions (Duncan et al. 2017; Ursache and Noble 2016), childhood adversity was associated with some potential stress-adapted skills that can be leveraged for positive outcomes (Ellis et al. 2022). Thus, further scrutiny was needed to clarify the complex association between childhood maltreatment and creativity.

### 4.2. The Mediation Role of Cognitive Flexibility

Consistent with our hypothesis, childhood maltreatment predicted individual creativity indirectly through cognitive flexibility. Previous research indicated that childhood maltreatment was associated with a range of cognitive function impairments and psychological disorders (Su et al. 2019; Xiao et al. 2021). Poor executive function was always found in adults with a history of childhood maltreatment or trauma (Letkiewicz et al. 2021). Recent surveys found that physical abuse and neglect usually led to a reduction in cognitive flexibility measured by CFI (Kalia et al. 2020), and early life stress negatively related to cognitive flexibility (Zhou et al. 2020). Another empirical study also indicated that both physical abuse and neglect were related with diminished cognitive flexibility as measured by the Wisconsin Card Sorting Test (WCST) (Spann et al. 2012). Childhood adversity was associated with lower cognitive flexibility, as measured by the Dimensional Change Card Sort (Zelazo 2006), relative to controls in children who lived in foster care (Lewis-Morrarty et al. 2012). Other studies further found that individuals who were exposed to early stress were impaired in cognitive flexibility measured by task-switching (Harms et al. 2018).

Cognitive flexibility is an important factor to influence creativity (Guilford 1967). Many studies focusing on cognitive control, attention, associative process, executive function, prefrontal function and REM sleep (Beaty et al. 2014; Dietrich 2004; Dorfman et al. 2008; Stickgold and Walker 2004; Zabelina and Robinson 2010) consistently indicated that cognitive flexibility was related closely to creativity. Furthermore, other studies also found cognitive flexibility was positively correlated with the performance of creativity measured by multiple dimensions of creativity, such as divergent thinking, convergent thinking, Chinese idiom riddle tests, and poster design tasks (Wu and Koutstaal 2020; Zuo et al. 2019). The present result further revealed that the cognitive flexibility measured by CFI positively predicted the self-report creativity as measured by WCAT. 

In line with the viewpoints of DPCM (Baas et al. 2013), childhood environmental factors influenced individual creativity through cognitive flexibility. Therefore, childhood maltreatment might cause lower cognitive flexibility. Consequently, poor cognitive flexibility further brings a low ability to shift thinking to adapt to the changing environmental stimulus, which might eventually lead to a reduction in creative ideas or innovations. That is, the effect of childhood maltreatment on creativity in college students might pertain through the mediation effect of cognitive flexibility.

### 4.3. The Mediation Role of Self-Efficacy

Additionally, childhood maltreatment also predicted individual creativity indirectly through self-efficacy. With higher self-efficacy, one has the belief that he/she has the ability to obtain a desired goal. Individuals who have experienced maltreatment or trauma are at increased risk for lack of the self-confidence that they can achieve remarkable success. Previous study indicated that undergraduates who experienced childhood emotional neglect generally formed the lower level of self-efficacy (Soffer et al. 2008). Similar results were also found in subsequent research (Lu et al. 2017). Social cognitive theory indicated that self-efficacy played an vital motivational effect in creativity performance (Bandura 1997). In addition, much empirical research showed that self-efficacy was associated positively with creativity. For example, a study on Grade 8-10 students found that self-efficacy was the first variable to explain the variance in creative emotion (Daher et al. 2021). Another paper enrolled 355 university students and revealed that there was a positive association between creativity and self-efficacy (Tamannaeifar and Motaghedifard 2014). The results of a meta-analysis research indicated that self-efficacy was positively related to self-rated creativity and divergent thinking (Haase et al. 2018). Thus, the earlier childhood maltreatment might lead to a low level of self-efficacy. Therewith, the lack of self-efficacy might contribute to doubt in one’s own abilities, which might finally lead to depressed creativity. It is probably fair to say that childhood maltreatment influenced creativity in college students through the mediation effect of self-efficacy.

### 4.4. The Serial Multiple Mediation Model

The present results showed that the association between childhood maltreatment and undergraduates’ creativity was a serial multiple mediation model. That is to say, earlier maltreatment experience indirectly influenced individual creativity through the effects of cognitive flexibility and self-efficacy. The above discussions indicated that childhood maltreatment was correlated with cognitive flexibility (Kalia and Knauft 2020; Spann et al. 2012) and self-efficacy was linked to creativity (Daher et al. 2021; Waheed and Dastgeer 2020). Bandura noted that the higher cognitive flexibility often brought the gradual accumulation of self-efficacy (Esen et al. 2017). Other empirical researches also indicated that there was a close relationship between cognitive flexibility and self-efficacy (Demirtaş 2020; Esen et al. 2017). Therefore, the earlier experience of maltreatment or trauma might induce the low level of cognitive flexibility. Consequently, the low level of cognitive flexibility might cause self-efficacy deficiency. Lastly, the insufficient self-efficacy might further lead to lack of creativity.

### 4.5. Limitations and Future Directions

There were some limitations of the present research that should be addressed. First, although the bootstrap process was used, the cross-sectional design and correlation analysis method could not clarify the causal relationship between childhood maltreatment and creativity and led to possible biased estimates of the parameters (Maxwell and Cole 2007). The serial multiple mediation model might need to be verified in further longitudinal research. Second, this research used a retrospective self-reported questionnaire to measure childhood maltreatment. There have been worries over whether participants could accurately report distal events occurring in childhood. Future research could take multiple forms of measures to assess earlier experience. Third, the present research only explored the relationship between childhood maltreatment and self-reported creativity and the mediation effects of cognitive flexibility and self-efficacy. Further research could clarify other potential factors that affect creativity, such as openness to experience (Takeuchi et al. 2012), intuition (Thomson and Jaque 2021) and schizotypal personality (Gibson et al. 2009), and other forms of creativity, for example, creative thinking and creative achievement.

## 5. Conclusions

In summary, although further validation and extension studies were needed, the present study was an important step forward in understanding how earlier maltreatment experience correlated with latter creativity. It indicated that cognitive flexibility and self-efficacy served as one potential mechanism by which childhood maltreatment was related to creativity. To better cultivate creative capacity and innovative talents, parents should give their children more love and care instead of neglect or abuse. Additionally, this research showed that the improvement of cognitive flexibility and self-efficacy would be more important in the future development of creativity performance.

## Figures and Tables

**Table 1:** Descriptive statistics results of study variables.

Variables	*M*	*SD*	Skewness	Kurtosis
Childhood maltreatment	42.38	13.73	1.31	0.54
Cognitive flexibility	66.82	8.52	−0.88	1.26
Self-efficacy	27.58	5.01	−0.12	0.73
Creativity	111.86	9.34	0.06	0.30

**Table 2:** Correlations among study variables.

	1	2	3	4
1. Childhood maltreatment				
2. Cognitive flexibility	−0.643 ***			
3. Self-efficacy	−0.547 ***	0.555 ***		
4. Creativity	−0.384 ***	0.447 ***	0.518 ***	

Note: *** *p* < 0.001.

**Table 3:** The serial multiple mediation effects of cognitive flexibility and self-efficacy between childhood maltreatment and creativity.

Path	Effect	SE	BootLLCI	BootULCI
Total effect (*c*)	−0.39 ***	0.03	−0.4459	−0.3322
Direct effect (*c’*)	−0.03	0.02	−0.0983	0.0417
*a1*	−0.64 ***	0.02	−0.6896	−0.5953
*a2*	−0.36 ***	0.03	−0.4186	−0.2938
*a3*	0.35 ***	0.03	0.2871	0.4102
*b1*	0.21 ***	0.04	0.1405	0.2786
*b2*	0.39 ***	0.03	0.3258	0.4536
Indirect effects				
Total indirect effects	−0.36	0.03	−0.4156	−0.3065
Indirect 1	−0.13	0.03	−0.1863	−0.0834
Indirect 2	−0.14	0.02	−0.1866	−0.1011
Indirect 3	−0.09	0.02	−0.1224	−0.0574

Note: *N* = 1069, *k* = 5000, *** *p* < 0.001. Indirect 1 = childhood maltreatment→cognitive flexibility→creativity; Indirect 2 = childhood maltreatment→self-efficacy→creativity; Indirect 3 = childhood maltreatment→cognitive flexibility→self-efficacy→creativity. BootLLCI = bootstrapping lower-limit confidence interval; BootULCI = bootstrapping upper-limit confidence interval; SE = standard error; Effect: standardized regression coefficient.

## Data Availability

The data are available on request to the corresponding author.
